# Determinants of nutritional status among adolescent tribal girls in Jharkhand: A grounded theory approach

**DOI:** 10.1016/j.dialog.2026.100303

**Published:** 2026-04-22

**Authors:** Litna George, Kumari Asha Kiran, Neelam Nalini, Manisha Kujur, Sunanda Jha, Amit Kumar, Arpita Rai, Ganesh Chauhan, Anit Kujur, Dewesh Kumar

**Affiliations:** a College of Nursing, Rajendra Institute of Medical Sciences, Ranchi, Jharkhand, India; b Community Medicine, Rajendra Institute of Medical Sciences, Ranchi, Jharkhand, India; c Department of OBG, Rajendra Institute of Medical Sciences, Ranchi, Jharkhand, India

**Keywords:** Nutritional determinants, Tribal adolescents girls, Grounded theory, India

## Abstract

**Purpose:**

Adolescence is a critical period of rapid growth where nutritional needs are heightened by biological and social factors. Tribal adolescent girls in rural India experience a disproportionate burden of undernutrition despite the presence of multiple government nutrition programmes. Existing research is largely quantitative and inadequately explains the social and institutional processes sustaining malnutrition. So this grounded theory explaining the determinants of nutritional status among adolescent tribal girls.

**Methods:**

This qualitative study used a constructivist grounded theory design conducted among ten government school teachers. Data was collected by in-depth face-to-face interviews in the month of April 2025. Collected data were analysed by using Open Code version 4.03, using constant comparison, memo writing, and theoretical integration following Charmaz's grounded theory approach.

**Results:**

The core process identified was *normalising gendered undernutrition through household survival practices and institutional non-responsiveness*. Four interrelated processes shaped this outcome: reproducing nutritional deprivation through gendered household practices; prioritising household survival over nutritional continuity; failing to translate institutional nutrition intentions into household practice; and adapting to nutritional deprivation through constrained agency and health avoidance. Together, these processes rendered undernutrition an ordinary and socially legitimate condition rather than a health crisis.

**Conclusion:**

Undernutrition among tribal adolescent girls is sustained through everyday adaptations shaped by poverty, gender norms, and weak institutional mediation. Addressing adolescent malnutrition requires interventions that break the social normalisation of deprivation, strengthen culturally relatable programme delivery, and engage household survival rationalities beyond expanding nutritional coverage alone.

## Highlights

• This grounded theory explains the determinants of nutritional status among adolescent tribal girls.
• Normalising gendered undernutrition through household survival practices and institutional non-responsiveness is the main reason for undernutrition
• Provide evidence to strengthen the current programmes and policies related to malnutrition and anaemia.

## 1 Introduction

Adolescence is an age group between 10 and 19 years that is a transitional phase from child to adult with rapid growth and development [1]. During this period, nutritional requirements increase drastically due to changes in dietary requirements, behaviours and environmental exposures [2]. Proper nutrition during this period not only helps the overall development but also makes a foundation for optimal health in the future [3]. Lack of proper nutrition during this period can lead to the triple burden of malnutrition, which includes undernutrition, overnutrition, and micronutrient deficiencies [2].

India, the home of 253 million adolescents; one in every five people is aged between 10 and 19 years [4]. Although the Indian economy improved over the years, malnutrition and anaemia continue to be the most significant issues [5]. In India, 53% of women aged 15–49 are anaemic [6], and the burden of thinness (BMI < 18.5 kg/m^2^) among adolescent girls of 15–19 years of age is 42% [7]. Similarly, the burden of thinness among adolescent girls of age 10–14 is 77%, and that of the 15–18-year-old age group is 45% [8], and people from rural areas and indigenous communities are more likely to experience malnutrition [9] than other communities.

Since independence, multiple programmes and policies have been implemented by the government, focusing on improving the education, health and livelihood of tribal communities. Despite the effort and special treatment, tribal communities are still the most nutritionally deprived segment of Indian society. The latest data shows that 4.7 million tribal children in India suffer from nutritional deprivation, which can lead to poor growth and development, as well as negatively impact their overall school performance and productivity in adulthood. On the other hand, stunting among under-five children is 40%, among which 16% are severely stunted. It is also found that mild and moderate stunting have a similar pattern in tribal and non-tribal children; severe stunting is higher among tribal children [10].

It is to be noted that 80% of the total chronically undernourished children from tribal communities in India come from eight states, one of which is Jharkhand, located in the eastern part of India. In the state, 6,642,982 non-pregnant women aged 15–49 are anaemic, while 2,638,835 are underweight [10].

Although the government of India has implemented many programmes and policies to tackle malnutrition, such as Integrated Child Development Services (ICDS), the National Health Mission, the Janani Suraksha Yojana, the Matritva Sahyog Yojana, the Midday Meal Scheme, the National Food Security Mission, etc. [11], malnutrition and anaemia are still concerns, although they have improved over the years.

There are many causes of dietary insufficiency among teenagers, including high growth requirements, eating patterns and lifestyles, risk-taking behaviours, and susceptibility to environmental influences [12]. In addition to that, poverty and inadequate socio-economic conditions, such as omitting breakfast, insufficient food diversity, cultural norms, and discrimination, as well as peer influence, can contribute to nutritional deficiencies [12]. Inadequate nutrition in adolescence can potentially retard growth and sexual maturation [12].

Prior to this study, several national surveys, such as the NFHS-5 (National Family Health Survey) [13] and studies by organisations like UNICEF [14] and ICMR, documented high rates of malnutrition, anaemia, and stunting among tribal adolescent girls in India. These studies primarily identified malnutrition through quantitative indicators such as BMI, haemoglobin levels, or dietary intake assessments. However, very few qualitative studies explored the underlying social, cultural, and systemic barriers contributing to these outcomes—especially from the perspectives of frontline stakeholders, such as schoolteachers.

This study is unique in its use of constructivist grounded theory to examine the determinants of nutritional status among adolescent tribal girls. While various models and theories, such as the social ecological model by UNICEF and the Dahlgren-Whitehead rainbow model for undernutrition among under-fives, have given advanced understanding about the determinants, they are insufficient to capture the determinants of malnutrition among adolescent tribal girls, as their food intake is influenced by various sociocultural factors that include traditional dietary practices, family roles in food preparation, and community beliefs about nutrition. This study adopts a grounded theory model that serves as an empirically grounded and locally relevant explanatory framework, aiming to generate a theoretical model that integrates educational, cultural, gender-based, economic, and programmatic dimensions of malnutrition, which may also contribute to achieving the sustainable development goals [10].

This study also provides a deeper contextual understanding of why existing nutritional interventions fail to produce expected outcomes among tribal adolescent girls; understanding the determinants of nutritional status among adolescent Indigenous girls residing in rural and backward areas helps maximise the controlling efforts.

## 2 Methods

### 2.1 Study design

The study used qualitative research approach with a design of constructivist grounded theory (CGT), developed by Charmaz In (2006). Constructivist grounded theory aims to generate a theory from a data while acknowledging the role of researcher in constructing and interpreting that data [15].

### 2.2 Researchers positioning

In constructivist grounded theory development, a theoretical framework is developed through the constant interaction between researchers and participants. Understanding the positioning of the researcher is essential. The principal investigator (LG) is an Indian nurse and academic currently positioned as a lecturer. During her MPhil in nursing, she was introduced to the topic of malnutrition, its prevalence and its causes. During her doctoral program, she further pursued the same topic, primarily focusing on indigenous teenage girls. As a lecturer closely associated with adolescent girls and the topic of malnutrition, the researcher initially interpreted the data as attributing blame to various systems that underplay the determinants of nutritional status among adolescent girls. Later, she acknowledged that her professional bias could influence this interpretation, which prompted her to re-examine the data.

### 2.3 Recruitment of the participants

A total of 10 samples (*n* = 10) were recruited to this study using a purposive sampling technique. The participants of the present study were school teachers and principals of various government schools in the Kanke block, Jharkhand. Because healthcare professionals rarely visit rural Jharkhand and parents and students are reluctant to share information, researchers selected school teachers, who interact with the adolescents on a daily basis and are the first ones to notice any changes in the adolescents' health, school performances, and attendance. Teachers, especially those working in rural areas, often visit students' households, and they know exactly what the socio-cultural factors, family circumstances, and school infrastructure that affect adolescent nutrition are. To mitigate the information distance from teachers' perspectives and perceptions, data were triangulated across different participants from different schools and participants from different roles.

### 2.4 Study setting

The study was conducted in Kanke block in Ranchi, Jharkhand state, an eastern state in India, which is predominantly a tribal one. The total tribal population in the state is 26.21% of the total population, with a total of 32 tribes. Some of the tribes are the Santhal, Munda, Kharia, Ho, and Oraon. This is a predominantly rural block, and people do agriculture and go to daily wage labour for a livelihood. This block has its own socio-economic challenges, such as a poor health care system, poverty, and cultural factors that cause nutritional vulnerabilities among children, which can lead to higher rates of malnutrition and related health issues in the community. Data were collected from 8 different middle and higher secondary government schools in the Kanke block. These centres were selected to achieve the maximum variation in the data.

### 2.5 Data collection

Data were collected in the month of April 2025 by in-depth face-to-face interviews. At first, a semi-structured interview guide was prepared based on the study objective. The guide included the core questions and some prompt questions. The interview guide was pretested and piloted among three school teachers from the same setting to determine feasibility. Minor modifications were done to the interview guide after the pilot study. The interview was held on the school premises in a quiet and separate room to ensure confidentiality and keep it away from all the distractions. Each interview lasted for 30 to 60 min with a median of 45 min. All the interviews were held by the principal investigator, a PhD scholar, and a lecturer with prior knowledge of qualitative research methodologies and interview techniques. Since this study was part of a mixed-methods study, the researcher was familiar with all the government teachers at each school, having been involved in this project since 2021, which facilitated the development of trust with the study participants.

Interviews were conducted primarily in Hindi and English based on the preference of the participants. After getting the participant consent, data was collected and audio was recorded. Later, audio was transcribed using Turbo AI and translated to English for making the initial manuscript. One participant who was not willing to give consent but verbally gave the information was not included in the study. Translation was done by the principal investigator but verified by another researcher who is fluent in both Hindi and English. Field notes were written during and immediately after the interview to document the non-verbal cues and contextual observations. Finally, the manuscript was verified against the audio recording multiple times to assess the accuracy. Before analysis, the final manuscript was anonymised. The final interview guide, including key questions and prompts, is provided in the supplementary material 1.

### 2.6 Data analysis

We analysed the data using Open Code version 4.03 [16]. Two researchers, LG and KAK, independently coded the entire transcript, resolving discrepancies through mutual discussion. Analysis of the data was done concurrently with data collection. We used constant comparison and reflexive interpretation for data analysis. In constructivist grounded theory, the sample size is determined by the depth, richness, and analytical sufficiency of the data. Theoretical saturation was assessed iteratively during data collection and analysis when no new data or codes emerged for major categories after the interview of participant number 10. Finally, an informal follow-up meeting was set up with selected participants to ensure that findings accurately reflected their experiences.

### 2.7 Initial (open) coding

After going through the data line by line, coding was performed to stay true to the participants' verbatim responses and actions. This process generated the initial 24 codes largely using gerunds from the included data. The formulated initial codes are as follows: lack of awareness about nutrition inadequate parental education, lack of parental involvement, children skipping nutritional supplements, junk food preference, not eating substitute food, poor hygiene practices, social stigma around menstruation, poverty and food insecurity, early marriage and adolescent pregnancy, limited access to a balanced diet, gap between rural and urban populations, and nutritional food being sold for income. Paternal alcohol misuse, disbelief of the people, personality trait of the girl child, gender-based discrimination, cultural beliefs and taboos, workload of the girl child, infrequent school attendance, limited health monitoring and school health activities, overburdened teachers and schools, ineffective implementation of government programmes and absence of localized health data. For example, the participants said people lacked awareness and knowledge of actual nutritious food. These codes were continuously compared within and across the data to show the differences, similarities, and variations of the experiences.

### 2.8 Focused coding and category development

Through the constant comparisons and analytical memo writing, conceptually related initial codes were clustered into eleven axial codes focused categories based on the shared properties and dimensions of the codes. It was analytical abstraction rather than reduction of the data. The reduction of 24 initial codes to 11 axial codes followed by four categories reflected the theoretical integration where codes with similar meanings are integrated into groups to provide conceptual clarity. For example, the initial codes, such as lack of awareness about nutrition, inadequate parental education, lack of parental involvement, and children skipping nutritional supplements, were integrated into the axial code of restricting access to actionable nutritional knowledge. Coding decisions were guided by questions such as ‘How is this code similar to other code?’ and ‘Under which category can this code be placed?’. Details are given in Table 1.

**Table 1:** Coding tree showing the progression from initial codes to axial codes and categories to the core category along with exemplar codes in the grounded theory analysis of teacher on determinants of malnutrition among adolescent tribal girls.

SL No	Initial codes	Axial codes	Categories	Exemplar quotes	Core category
1	Gender-based discrimination Workload of the girl child Social stigma around menstruation Cultural beliefs and taboos	Assigning gendered labour while deprioritising girls' nutrition	Reproducing nutritional deprivation through gendered household practices	“At home, brothers and sisters… there is a difference between a boy and a girl… Girls will work more, and boys will rest… Boys will eat more, and girls will eat what is left.” (P2)	Normalising Gendered Undernutrition through Household Survival Practices and Institutional Non-Responsiveness
2	Early marriage and adolescent pregnancy	Transferring nutritional vulnerability across generations through early marriage		“In the rural areas, the girls hardly remain in the houses… Early marriage also… They don't get sufficient nutritional value and health… The girl becomes a mother at the age of 15.” (P3)	
3	Paternal alcohol misuse	Redistributing household burdens onto women amid substance-related disruption	Prioritising household survival over nutritional continuity	“Most of the times, they involve themselves in Hariya drinking… They remain idle, and they don't work in a day.” (P3)	
4	Poverty and food insecurity Nutritional food is being sold for income	Allocating food resources based on survival hierarchies under economic uncertainty		“In government school, most of the kids come from poor backgrounds… The parents cannot give everything… If their father is not there, then the child will have to work just to earn for a day.” (P1)	
5	Lack of parental involvement Infrequent school attendance	Withdrawing parental nutritional oversight amid livelihood pressures		“We have PTM once a month… difficultly only 2–5 people come… they listen and go. But they do not follow it.” (P7)	
6	Lack of awareness about nutrition Inadequate parental education Limited access to balanced diet	Restricting access to actionable nutritional knowledge	Failing to translate institutional nutrition intentions into household practice	“We don't have to tell the urban areas. Because they used to google everything. But, in rural areas, they are not googling. They are not aware enough to google.” (P1)	
7	Disbelief of the people Gap between rural and urban population	Alienating communities through linguistic and cultural misalignment		“They lack in expressing their own viewpoint… They feel shy because they are not confident enough in their national language or the state language.” (P3)	
8	Children skipping nutritional supplements Not eating substitute food	Avoiding formal nutritional interventions amid fear and mistrust		“There are some children who are afraid… What medicine is being given here? … Some children get scared, some become unconscious, some start crying… Some put it in their mouth and throw it.”	
9	Poor hygiene practices Limited health monitoring and school health activities	Normalising everyday health risks as unavoidable	Adapting to nutritional deprivation through constrained agency and health avoidance	“They don't use sanitary pads; they use cloth… If they use cloth for more than 8 h, then it has a detrimental effect on our body.” (P2)	
10	Junk food preference Personality trait of the girl child	Silencing adolescent nutritional needs while negotiating dietary transition		“Girls are shy because they are the newly grown-up child… When they grow up, then only they can understand this is an issue.” (P3)	
11	Ineffective implementation of government programs Overburdened teachers and schools Absence of localized health data	Performing policy commitment without operational follow-through		“The government is doing its job. They are giving food… I don't want to talk much about the quality of the food.” (P1)	

Notes: Initial codes are generated by going through the manuscript line by line, following the progression of axial codes. Categories were developed by constant comparison and memo writing. Participants were school teachers, and the interview was conducted in both Hindi and English.

### 2.9 Theoretical integration

The emergent categories were refined through constant comparison with new data. The relationship among categories was explored to find out the core explanatory process to find out the determinants of nutritional status among adolescent tribal girls. Data collection continued until theoretical sufficiency was achieved, whereby additional data no longer contributed substantively new properties to the emerging categories. The core category of the theory, which focusses on normalising gendered undernutrition through household survival practices and institutional non-responsiveness, is connected to subcategories that include reproducing nutritional deprivation through gendered household practices, prioritising household survival over nutritional continuity, failing to translate institutional nutrition intentions into household practice, and adapting to nutritional deprivation through constrained agency and health avoidance. We formed the final theory by continuously comparing the differences and similarities in the data analysis. Memo writing took place throughout the study. Member checking was conducted with five participants to validate the emerging categories.

### 2.10 Rigour and trustworthiness

#### 2.10.1 Credibility

The study maintains its methodological rigour by adhering to the guidelines provided by Lincoln and Guba [17]. The credibility of the data was ensured by maximising sample variability, engaging with the data over an extended period, conducting continuous observations, maintaining referential adequacy, and writing iterative memos. Peer debriefing was conducted with an experienced researcher in this field during the initial coding, category formation, and theoretical development phases.

#### 2.10.2 Dependability

The dependability of the study was ensured through an audit trail of the data, documentation of data collection and analysis procedures, and continuous monitoring by two expert researchers. Negative cases were examined throughout the analytic process. The categories which are not fitting to the emerging categories are examined in detail. These negative cases were compared with previously coded data to ensure the analytical depth and refine the conceptual categories. Negative case analysis also helped to modify and give better clarification of the emerging categories. This process strengthens the explanatory power of the emerging grounded theory by including both convergent and divergent participant experiences.

#### 2.10.3 Transparency

Transparency was maintained throughout the procedures, whereas ambiguity was resolved amicably. We supported transferability by providing detailed descriptions of the subjects and conducting the study in eight different centres. Despite the inability to perform formal inter-rater reliability, we maintained analytical rigour by relying on peer validation of interpretive coherence.

### 2.11 Ethical considerations

The study received approval from the Institutional Ethical Committee at the Rajendra Institute of Medical Sciences. This study is part of a mixed-method research project titled “Determinants of Nutritional Status and the Effectiveness of the Malnutrition Treatment Module for the Prevention and Control of Malnutrition Among Young Adolescent Tribal Girls of Ranchi, Jharkhand.” The study was also registered under CTRI (CTRI/2024/05/066759). Informed written consent in English was obtained from all the participants. Additionally, participants were informed that their audio would be recorded, but their personal details would remain confidential. All the recorded data was kept confidentially on the principal investigator's password-protected laptop. Identification details of the participants were removed and replaced with codes.

## 3 Results

### 3.1 Description of sample characteristics

Most of the participants included in this study were aged more than 30 years. All of them were males except one participant. Seven participants had postgraduation. Six of them had more than 10 years of teaching experience. Among all the participants, two of them were working as the principals. All the participants were working in middle school except two teachers; they were working at the secondary level. All the teachers were working in the rural areas (Table 2).

**Table 2:** Description of sample characteristics based on the socio demographic variables of school teachers from government schools.

Participant ID	Role	Sex/Gender	Age group	Years of experience	School level	School location
P1	Teacher	Male	40–49	15	Middle School	Rural
P2	Principal	Male	50–59	27	Higher Secondary	Rural
P3	Teacher	Male	40–49	18	Middle School	Rural
P4	Teacher	Male	30–39	10	Middle School	Rural
P5	Teacher	Female	20–29	2	Middle School	Rural
P6	Principal	Male	50–59	20	Middle School	Rural
P7	Teacher	Male	20–29	2	Middle School	Rural
P8	Teacher	Male	40–49	16	Middle School	Rural
P9	Teacher	Male	40–49	9	Middle School	Rural
P10	Teacher	Male	30–39	7	Higher Secondary	Rural

*Notes**:*** Participants were school teachers working in the various government schools in Kanke Block in Ranchi, Jharkhand, an eastern state in India. Identifiers were used to maintain confidentiality. Age and years of experience were reported at the time of data collection.

### 3.2 Formulation of grounded theory

A total of four categories: reproducing nutritional deprivation through gendered household practices, prioritising household survival over nutritional continuity, failing to translate institutional nutrition intentions into household practice and adapting to nutritional deprivation through constrained agency and health avoidance emerged from 11 axial codes such as assigning gendered labour while deprioritising girls' nutrition, transferring nutritional vulnerability across generations through early marriage, redistributing household burdens onto women amid substance-related disruption, allocating food resources based on survival hierarchies under economic uncertainty, withdrawing parental nutritional oversight amid livelihood pressures, restricting access to actionable nutritional knowledge, performing policy commitment without operational follow-through, alienating communities through linguistic and cultural misalignment, avoiding formal nutritional interventions amid fear and mistrust, normalising everyday health risks as unavoidable and silencing adolescent nutritional needs while negotiating dietary transitions. In the end, the core category was formed: “normalising gendered undernutrition through household survival practices and institutional non-responsiveness.” were created from the axial codes that relate to the main category, which helped build the theoretical framework on what affects nutritional status. The following were the axial codes that emerged from this study.

#### 3.2.1 Restricting access to actionable nutritional knowledge

Teachers described how the lack of nutrition among adolescent tribal girls is not only due to food scarcity but also due to restricted nutritional information circulated within the family and community. Despite the availability of locally nutritious foods such as ragi and bajra, parents and children are often unaware of how to use them and their nutritional value. This lack of awareness leads to the underutilisation of locally available food by adolescent tribal girls, which could otherwise support their nutrition.

One teacher from the school, situated deep inside a rural area surrounded by forest, says the underutilisation of locally available food is due to a lack of knowledge.

“Probably, you must know, many of the parents… You have heard about ragi, which we call here madwa… People were not aware of it. Right now, in urban areas, you will see that most of the people are buying that… However, they are unaware of what constitutes actual nutritious food.” (P1).

This restricted awareness is further exacerbated by information isolation. Rural residents lack access to digital or other health-related information. Teachers contrasted rural contexts with urban settings, noting that parents and adolescents in rural communities seldom accessed online and offline information to supplement school-based health education.

“We don't have to tell the urban areas. Because they used to google everything. But, in rural areas, they are not googling. They are not aware enough to google.” (P1).

Even when teachers attempt to provide nutritional education and guidance, it often goes in vain due to limited parental engagement and weak reinforcement at home. So, the nutritional education has failed to translate to everyday practice, leading to malnutrition among adolescent girls.

#### 3.2.2 Allocating food resources based on survival hierarchies under economic uncertainty

Teachers described poverty not as the scarcity of money but as a structural condition that intervenes in everyday food practice. Due to economic uncertainty, parents prefer immediate survival needs rather than food quality, resulting in persistent food insecurity among adolescent girls.

A teacher working in the remote area school explains how economic constraints lead parents to not provide nutritionally adequate meals to children.

“In government school, most of the kids come from poor backgrounds… The parents cannot give everything… If their father is not there, then the child will have to work just to earn for a day.” (P1).

This economical pressure is leading to survival-driven food allocation. People, especially from remote areas, usually have monotonous foods dominated by rice, limited to boiled potato and dal along with rice, and limit their diet to twice a day. Teachers noted that nutritionally valuable food items are usually sold to meet immediate cash needs, whereas the less nutritious and damaged food items are retained.

“He produces crops… He keeps the bad items at home. He sells the good items… the reason for this is poverty.” (P9).

Through this process food is taken away from the household, leading to reduced food diversity and nutritional intake. Adolescent girls' nutritional requirements start during this period, particularly affecting resulting nutritional vulnerability.

#### 3.2.3 Assigning gendered labour while deprioritising girls' nutrition

Teachers described how gender and cultural norms are not merely a belief but a practice that systematically shapes the food distribution and workload within the household. Adolescent girls are burdened with all the household work while receiving comparatively less food than boys.

One participant explained how these norms operated routinely within families:

“At home, brothers and sisters… there is a difference between a boy and a girl… Girls will work more, and boys will rest… Boys will eat more, and girls will eat what is left.” (P2)

These gender-based differences resulted in nutritional deficiency among adolescent girls. Over the period, this has become normalised and socially accepted. As a consequence, malnutrition among adolescent girls is considered an expected outcome in households.

#### 3.2.4 Performing policy commitment without operational follow-through

Teachers described government programmes related to nutrition and health as available, but they are implemented weakly, resulting in limited benefits for adolescent girls. Schemes such as midday meals up to 8th standard, iron and folic acid supplementation, the recently started ragi laddu supplementation and school health check-ups are active in every school. Their impact was affected by a lack of monitoring and resources and improper implementation at the school level.

One teacher acknowledged the provision of midday meals but showed concern about the quality of the food.

“The government is doing its job. They are giving food… I don't want to talk much about the quality of the food.” (P1).

Reduced financial allocation to midday meals is also affecting the quality of the food and food diversity, limiting the inclusion of nutritious food and compromising the quality of food.

“I am giving them midday meals… It would be better to give them some extra milk.” (P6).

Apart from the quality of food, teachers also highlight the irregular implementation of health check-ups, including periodic measurement of anthropometry, eye, dental and anaemia check-ups.

“The whole check-up is done… but it is not that it happened every year.” (P6).

Teachers also expressed logistic barriers, such as students not consuming eggs, the provision of giving fruits, and implementation challenges in rural and remote areas.

“Fruits are not given… to get fruits from here, we have to go so far.” (P9).

Teachers also described how schools do not have sufficient teachers to monitor the health-related activities of the school, and staff in school are being overburdened with academic and other administrative activities.

“The government is trying, but schools are not able to do this because we have so many programmes.” (P5).

“There are only two teachers… from KG to A, it is difficult.” (P9).

Overall, the government programmes are functioning as institutional intentions rather than effective interventions, so it is limiting its intentions to compensate for household food insecurity and gender-based discrimination at home, leading to nutritional vulnerability.

#### 3.2.5 Avoiding formal nutritional interventions amid fear and mistrust

Teachers described supplementation of iron, folic acid and deworming tablets instilling fear and mistrust among children and parents that leads to the programme implementation being weak and less successful. Although these tablets are supplied from the school regularly, the response of the children is largely due to misconceptions about the medicine and fear of the medicine given from the external sources.

One teacher explained how the children are looking at the medicine as threatening rather than protective.

“There are some children who are afraid… What medicine is being given here? … Some children get scared, some become unconscious, some start crying… Some put it in their mouth and throw it.” (P7).

This fear towards medicine is leading the students to resistance. Some students are taking the tablets in front of the teachers; when they ensure the teachers are not observing, they are spitting them out. Some students are deliberately not coming to the schools when tablets are distributed.

“There are some children who do not come to school on the day when they think that they will get the medicine.” (P7).

Despite the best effort from the teachers, nutritional supplements are not yielding their full result due to a lack of trust among the students. It is also leading the adolescent girls to be more nutritionally vulnerable.

#### 3.2.6 Withdrawing parental nutritional oversight amid livelihood pressures

Teachers feel that disengagement from parents leads to a discontinuity of nutrition between school and home. Parent-teacher meetings are routinely arranged, but they are often unsuccessful because fewer parents participate, which restricts their knowledge acquirement on nutrition, health-related activities, and government welfare programmes.

“We have PTM once a month… difficultly only 2–5 people come… they listen and go. But they do not follow it.” (P7).

This phenomenon is resulting in the breakdown of knowledge reinforcement. Whatever children learn from the school is not translated to practices. Despite the institution's efforts, dietary practices remain largely unchanged.

In addition, irregular school attendance is also intensifying the existing malnutrition, as students are taking long absences from the school, especially during harvesting and cultivation season. The students from the economically weaker backgrounds were deprived of nutrition, as school is the gateway for daily nutritional support through midday meals. Long absenteeism therefore leads to nutritional deprivation among children.

“That one week, he didn't get the nutritious food that was available in the school. He didn't get it at home either.” (P1).

Together, parental disengagement with long absences leads to interruptions in nutritional continuity, resulting in deprived nutrition among adolescent girls.

#### 3.2.7 Transferring nutritional vulnerability across generations through early marriage

Teachers conceptualised early marriage and pregnancy, prematurely pushing adolescent girls from growth to reproductive roles, therefore interrupting their growth. Adolescent girls in the rural and interior areas often get married before their legal age, before achieving adequate physical and mental growth.

One participant explained how early marriage is common in rural areas and what their nutritional condition is:

“In the rural areas, the girls hardly remain in the houses… Early marriage also… They don't get sufficient nutritional value and health… The girl becomes a mother at the age of 15.” (P3).

The early pregnancy and motherhood lead to nutritional compromise among the adolescent girls, where the girls' growth needs compete with pregnancy needs. As a result, the adolescent girls entered the pregnancy with compromised health needs, leading to their own compromised health along with the risk of low birth weight and intergenerational malnutrition.

Through this process early marriage and pregnancy not only cause nutritional vulnerability among adolescent girls but also act as a mechanism for intergenerational malnutrition.

#### 3.2.8 Alienating communities through linguistic and cultural misalignment

Teachers describe that language differences are not merely communication barriers, but they are also leading to community disengagement with schools and health services. In Jharkhand, although Hindi is an official language for administration, people from different tribal groups use different languages; so many parents and students are more fluent in local languages rather than Hindi. This language barrier constrains their ability to articulate their health concerns, ask questions related to health and obtain clarification.

One participant explained how limited linguistic confidence translated into withdrawal:

“They lack in expressing their own viewpoint… They feel shy because they are not confident enough in their national language or the state language.” (P3).

This language barrier is leading the people to become passive recipients instead of active participants in health and health-related activities and programmes.

In addition to the language barrier, the teachers also described the village having a broader mistrust of the external systems and outsiders, which further reduces the uptake of the services. A teacher described an incident when a medical team visited the village for a health check-up; not a single person turned up from the village due to fear and disbelief. The team waited for two days; finally, they left the village.

“The medical team came… People did not come to take any medicine… the team left.”

This pattern shows the institutional distancing when there is fear and a lack of trust among the people in external people and the healthcare system and healthcare interventions. The combination of the linguistic barrier and poor acceptance of healthcare programmes is leading to limited access to nutritional and health support, which further exacerbates malnutrition.

#### 3.2.9 Normalising everyday health risks as unavoidable

Teachers explained that lack of cleanliness among adolescent girls is a normal process triggered by a lack of knowledge, awareness, and resources. The girls' menstrual hygiene practices are constrained; they use cloth instead of pads and also for long periods without changing it.

One participant explained how these practices were embedded in daily routines:

“They don't use sanitary pads; they use cloth… If they use cloth for more than 8 hours, then it has a detrimental effect on our body.” (P2).

This behaviour leads to increased vulnerability to infection among adolescent girls and also acts as an indirect contribution to malnutrition and health deterioration.

In addition to that, teachers also described using unsafe drinking water from wells, ponds, and rivers without boiling it.

“They use the water of the well, the river, the pond… they drink water without boiling it.” (P10).

The use of unsafe drinking water and poor menstrual hygiene also indicates the risk of normalising these practices among people. Together it leads to a hidden burden of health risk and nutritional vulnerability among adolescent girls.

#### 3.2.10 Redistributing household burdens onto women amid substance-related disruption

Teachers described alcoholism among households as not only an individual-level behaviour but also a household-level process that destabilises the economic security at the household. Daily consumption of locally brewed alcohol is also diverting household income away from food and other essential items needed for the household.

One participant described this recurring pattern:

“Most of the times, they involve themselves in Hariya (traditional rice beer) drinking… They remain idle, and they engaged with irregular wage labour in a day.” (P3).

As men are not doing caregiving and productive roles, women are forced to do multitasking jobs, including income generation and household chores.

“A lady has to work hard… She has to earn also, and then she has to look after her family also.” (P3).

This process leading to a care deficit within the households where the mother is going outside for earning money means adolescent girls are increasingly drawn to household chores. Finally, she utilises less time for studying and taking care of herself. Although alcoholism is not a direct influence on nutritional status, it is an indirect contributor to nutritional vulnerability among adolescent girls.

#### 3.2.11 Silencing adolescent nutritional needs while negotiating dietary transitions

Teachers describe the shyness of adolescent children as not inherited; rather, it results from various sociocultural factors that limit adolescent girls' ability to express their health and nutrition-related concerns.

One participant explained this hesitation as part of the transition into adolescence:

“Girls are shy because they are the newly grown-up child… When they grow up, then only they can understand this is an issue.” (P3).

The adolescent is also switching their food habits from traditional food to available ultra-processed food, which does not have any nutritious value. Teachers observed that parental practices often reinforce food preferences shaped early in childhood.

“There is a lot of junk food… parents are attracting them to junk food, rather than healthy food.”(P4).

Together with limited adolescent voice and normalisation of consumption of junk food, this contributed to suboptimal intake of nutritious food during adolescence, the critical period of growth and development, leading to undernutrition among adolescent girls.

### 3.3 Core process: Normalising gendered undernutrition through household survival practices and institutional non-responsiveness

#### 3.3.1 Theoretical proposition

Normalising gendered undernutrition through household survival practices and institutional non-responsiveness is the core process shaping the nutritional status of the adolescent girls. Undernutrition among adolescent girls arises from repeated social processes characterised by reproducing nutritional deprivation through gendered household preferences, where boys are favoured over girls, and nutritious food items are often sold out due to household survival practices over nutritional continuity. Institutions fail to translate policies and programmes aimed at improving the nutritional status of adolescent girls into culturally understandable and trusted practices, ultimately leading to adaptations that reinforce nutritional deprivation through limited agency and health avoidance, which normalises undernutrition as an ordinary and unavoidable condition.

This core process was constituted through four interrelated categorical processes:

(1) reproducing nutritional deprivation through gendered household practices;
(2) prioritising household survival over nutritional continuity;
(3) failing to translate institutional nutrition intentions into household practice; and.
(4) adapting to nutritional deprivation through constrained agency and health avoidance.

**Fig. 1:**
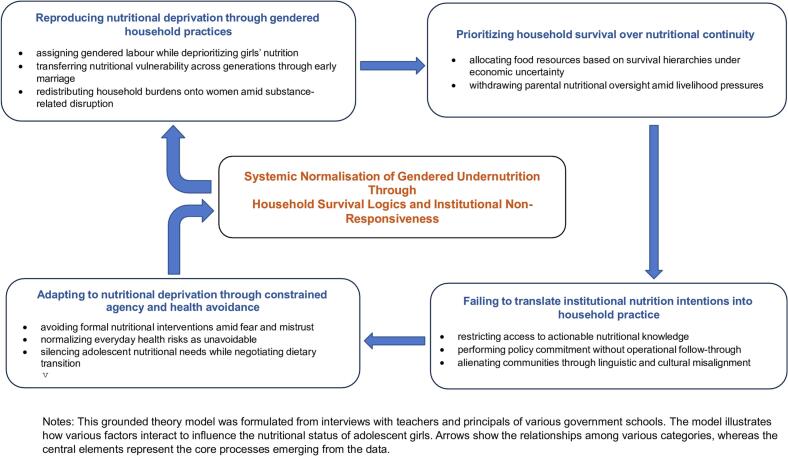
The grounded theory shows how the undernutritions are interrelated to each other.

These four processes show how undernutrition is interrelated to each other and it is a result of complex social process (Fig. 1).

#### 3.3.2 Reproducing nutritional deprivation through gendered household practices

Under conditions of entrenched gender norms and economic precarity, households reproduce nutritional deprivation through gendered moral expectations that structure labour, care, and food allocation. Carers interpreted girls' bodies as adaptable and resilient, legitimising the assignment of gendered labour while deprioritizing the girls' nutritional needs. This process was not framed by participants as neglect but as a reasonable adjustment to household realities.

Early marriage, on the other hand, acts as the mechanism that transfers nutritional vulnerability across the generation. As marriage is considered a solution for economic deprivation, girls are embracing motherhood before their growth and development are completed; as a result, there is a severe compromise of nutrition among adolescent girls within socially accepted life trajectories. Alcoholism-related household disruption further intensifies this process, shifting the caregiving and labour responsibilities onto women and adolescent shoulders, constraining the opportunities for consistent nutrition-related care.

Through this process undernutrition is considered as an ordinary outcome of gendered organisation rather than needing an intervention.

#### 3.3.3 Prioritising household survival over nutritional continuity

Due to economic constraints, most households prioritise immediate survival over nutritional continuity. As a result, nutritious food is being sold for economic benefit, whereas nutritional needs are considered secondary and negotiable. As carers feel nutritional inadequacy is less important than daily survival, it leads to compromising the nutritional practices and redefining demand from a sustained responsibility into an intermittent aspiration, depending upon surplus rather than need.

Prioritising household survival over nutritional continuity leads to normalised nutritional compromise within households.

#### 3.3.4 Failing to translate institutional nutrition intentions into household practice

Institutional nutrition programmes entered this context as symbolic commitments rather than operationally embedded practices. Participants described nutrition initiatives as present in policy discourse but weakly translated into household realities. Linguistic and cultural misalignment between service providers and communities restricted access to actionable nutritional knowledge, producing confusion rather than empowerment.

Institutional interactions were frequently interpreted through mistrust, as inconsistent delivery, poor follow-up, and limited engagement alienated caregivers and adolescents. As a result, institutional nutrition was constructed as external, unreliable, and misaligned with everyday survival priorities. Rather than compensating for household constraints, institutional non-responsiveness reinforced household withdrawal from formal nutritional support.

Through this process institution also unknowing normalising the malnutrition among adolescent girls.

#### 3.3.5 Adapting to nutritional deprivation through constrained agency and health avoidance

Avoiding formal nutritional interventions amid fear and mistrust, normalising everyday health risks as unavoidable and silencing adolescent nutritional needs while negotiating dietary transitions.

Within the convergence of household survival pressures and institutional non-responsiveness, adolescents and caregivers adapted to nutritional deprivation through constrained agency. Avoiding formal nutritional interventions amid fear and mistrust emerged as a pragmatic strategy to minimise perceived risks associated with unfamiliar or poorly explained programmes.

Everyday health risks were normalised as unavoidable, and adolescent nutritional needs were frequently silenced during dietary transitions marked by increasing consumption of low-quality or junk foods. Rather than expressing resistance, girls navigated deprivation quietly, negotiating food preferences within narrow boundaries of choice.

These adaptive practices did not eliminate agency but redefined it as survival-oriented adjustment, enabling households and adolescents to cope within structurally limited environments while further embedding undernutrition as a normal condition.

Together, these four processes interacted recursively. Gendered household practices and household survival closely interact with each other, which is further exacerbated by the institutional-level failure to translate institutional nutrition intentions into household practice. Through constrained agency and health avoidance, undernutrition became progressively normalised, keeping the core process of normalising gendered undernutrition through household survival practices and institutional non-responsiveness.

Undernutrition, therefore, is perpetuated not only by programmatic failure but also through a socially legitimate practice of adaptation and prioritisation co-constructed by household practices and institutions under structural constraints.

## 4 Discussion

The constructivist grounded theory developed in this study explains how the development of malnutrition among adolescent tribal girls becomes a normalised process. Rather than finding malnutrition as an individualised process, this theory conceptualises malnutrition as the outcome of the interaction of various factors such as gendered household practices, survival rationalities, and institutional disengagement. This study gives an extension of current nutritional studies by giving a process-related explanation of nutritional deprivation among adolescent girls.

### 4.1 Undernutrition as a normalised social process

Much of the nutrition literature conceptualises adolescent undernutrition because of poverty, food insecurity, or inadequate programme coverage [18], [19]. While these frameworks identify important structural determinants, they often under-theorise how deprivation is sustained in everyday life. The present grounded theory contributes by demonstrating that undernutrition persists not simply because resources are scarce, but because deprivation is rendered ordinary, expected, and morally acceptable within households facing chronic precarity. This study shows that gendered undernutrition is not experienced as a crisis demanding an urgent response. Instead, it is absorbed into routine household functioning, where girls' nutritional compromise is interpreted as adaptive rather than harmful.

### 4.2 Gendered household practices and survival rationalities

The theory explains how gendered household practices shape the nutritional outcome among adolescent girls. Assigning more households and other work to girl children while deprioritising their nutrition, transferring vulnerability through early marriage, and redistributing household burdens amid alcohol-related disruption reflect patterns documented in various research studies [20]. This study advances the literature by theorising these practices not as isolated cultural norms, but as a household rationality.

Households prioritise immediate survival over nutritional continuity, in line with findings from livelihood and food security studies that show how scarcity restructures care priorities. Importantly, parents did not perceive these decisions as neglectful; rather, they framed them as necessary for survival. This theory challenges the existing behaviour models that attribute adolescent malnutrition to sheer ignorance or an attitude from the parents or caretakers, underscoring the need to understand nutritional compromise as a socially legitimate adaptation.

### 4.3 Failing to translate institutional nutrition intentions into household practice

A critical contribution of this study lies in theorising the role of institutions not merely as absent or weak, but it is not actively translating institutional nutrition intentions to household practices. While implementation research frequently highlights gaps between policy and practice, the present theory shows how linguistic and cultural misalignment, performative policy commitment, and inconsistent engagement produce mistrust and withdrawal, rather than compliance.

### 4.4 Constrained and adaptive agency

Girls avoid nutritional intervention because they fear and mistrust the normalisation of everyday health risks, and their inability to express their nutritional needs results in nutritional compromise. This extends the current literature, as underutilisation of nutritional interventions and services as resistance or lack of awareness to avoidance functioned as risk management, reflecting a rational response to poorly understood institutional interventions. Programmes such as Anaemia Mukt Bharat aim to address anaemia by providing iron and folic acid supplementation to children until the 8th standard, but the participants observed that weak programme delivery and mistrust among the children of outsiders and supplements from outside prevent them from getting the full potential of the programmes. Similarly, midday meal is also provided to children until middle school, although it supports the nutritional needs of the children; the participants observed compromised food quality and lack of a monitoring system. This insight has important implications for program design; it suggests that increasing coverage alone is insufficient without addressing the relational and interpretive dimensions of care.

The findings of this grounded theory study suggest that malnutrition among adolescent tribal girls is not solely the result of individual or household behaviours but emerges through the interaction of structural deprivation, weak institutional mediation, and constrained adolescent agency. So, addressing undernutrition among adolescent tribal girls is needed—disrupting the social process that normalises gendered undernutrition rather than correcting the dietary intake or expanding the program's reach. By providing evidence on determinants of nutrition among adolescent indigenous girls, this study also contributes to achieving sustainable development goals 2, 3, 5 and 10 that emphasise no hunger, good health and wellbeing, gender equality, and reducing inequalities [10].

The theory also enlightens policymakers, healthcare workers, and researchers about the mechanisms that generate and sustain undernutrition. So, the proposed interventions should be responsive, rational, and grounded in social and cultural sensitivity, recognising that trust, legitimacy, and continuity are as critical to reducing the nutritional vulnerability.

This theory proposes policies needed to engage household survival rationalities and interventions needed to focus on the prevention strategies for gendered labour and care expectations so that intergenerational vulnerability to malnutrition can be reduced. The study also proposes culturally accepted education programmes for adolescent girls and their caretakers to facilitate the delivery of various nutritional initiatives. Constant monitoring and implementation of various nutrition-related programs may help improve nutritional status. Community participation, which includes adolescent girls, their parents, and other community members, may increase the acceptance of various programmes and change the community's perception of nutrition for adolescent girls.

## 5 Strength and limitations

This is the first study conducted in India to find out the determinants of nutritional status among adolescent tribal girls using grounded theory. This study takes the perspectives of the school teachers who have knowledge of ground reality. This theory offers a process-oriented explanation of malnutrition among adolescent girls and, at the same time, provides some solutions.

The study is limited because it relies solely on the perspectives of schoolteachers and does not include the voices of other stakeholders, such as adolescent girls, parents, or community members, which prevents it from capturing the exact reasons for malnutrition at the individual, family, and community levels, resulting in a theory that is contextually bounded. Additionally, we included the maximum number of male participants. This reflects staffing patterns; gender-sensitive issues were nevertheless explicitly explored and may have influenced the interpretation of nutritional status from the viewpoint of a woman. The finding should therefore reflect the viewpoint from the institutional level rather than from the perspective of adolescent girls lived experiences. Future research incorporating adolescent girls, parents, and community stakeholders in adolescence will yield a better perspective on this problem. Despite the researcher's use of reflexive strategies, her bias may have influenced the study's interpretation. The authors recognise the ethical risk that research documenting deprivation may inadvertently reinforce deficit-based stereotypes about tribal communities. This study does not interpret practices such as early marriage, dietary compromise, or programme disengagement as inherent cultural characteristics. Rather, these emerged as contextually situated responses to structural poverty, gendered constraints, and prolonged institutional non-responsiveness. By giving emphasise on lived experiences and social processes, the study seeks to challenge, rather than reproduce, narratives that marginalise tribal populations.”

## 6 Conclusion

This study develops a constructivist grounded theory showing how undernutrition among adolescent girls becomes normalised through the interaction of household survival practices and institutional non-responsiveness. Undernutrition among adolescent tribal girls is shaped not only by individualised limitations but also by socially legitimate adaptations shaped by gendered household organisation and survival rationalities. Whereas the institution is failing *to translate institutional nutrition intentions into household practice,* limited due to linguistic barriers and mistrust and flawed programmatic implementation. By conceptualising constrained agency and health avoidance as adaptive responses rather than neglect, this study advances a process-orientated understanding of persistent nutritional inequities and highlights the need for intervention in the social and institutional conditions that lead to persistent undernutrition.

## Declaration of generative AI in manuscript preparation

“During the preparation of this work, the authors used TurboScribe.ai to transcribe audio-recorded interviews. The authors also used QuillBot to edit the grammar. The transcripts were reviewed and edited by the authors for accuracy, and the authors take full responsibility for the content.”

## CRediT authorship contribution statement

**Litna George:** Writing – original draft, Software, Resources, Methodology, Investigation, Formal analysis, Data curation, Conceptualization. **Kumari Asha Kiran:** Conceptualization, Methodology, Validation, Formal analysis, Investigation, Resources, Data curation, Writing – original draft, Writing – review & editing, Visualization, Supervision, Project administration. **Neelam Nalini:** Writing – review & editing, Validation, Supervision, Project administration, Methodology, Investigation, Formal analysis, Conceptualization. **Manisha Kujur:** Writing – review & editing, Validation, Supervision, Resources, Project administration, Conceptualization. **Sunanda Jha:** Writing – review & editing, Validation, Supervision, Project administration. **Amit Kumar:** Writing – review & editing, Validation, Supervision, Resources, Project administration. **Arpita Rai:** Writing – review & editing, Validation, Supervision, Resources, Project administration. **Ganesh Chauhan:** Writing – review & editing, Validation, Resources, Project administration. **Anit Kujur:** Writing – review & editing, Validation, Supervision. **Dewesh Kumar:** Writing – review & editing, Validation, Supervision, Project administration.

## Ethical approval

The study received approval from the Institutional Ethical Committee at the Rajendra Institute of Medical Sciences, Ranchi, Jharkhand.

## Funding

This research is not received any specific grant from any funding body.

## Declaration of competing interest

The authors declare the following financial interests/personal relationships which may be considered as potential competing interests:

**Table:** 

Litna George reports administrative support was provided by Rajendra Institute of Medical Sciences. Litna George reports a relationship with Rajendra Institute of Medical Sciences that includes: employment. Litna George has patent NIL pending to Not applicable. NIL If there are other authors, they declare that they have no known competing financial interests or personal relationships that could have appeared to influence the work reported in this paper.

## Data Availability

Due to the sensitivity of the topic, data will be kept as confidential and will not be shared in public.

## Appendix A Supplementary data

Supplementary material 1 Interview guide
