# Supplementary material for: Determinants of nutritional status among adolescent tribal girls in Jharkhand: A grounded theory approach
Source: Dialogues Health. 2026 Apr 22;8:100303. doi: 10.1016/j.dialog.2026.100303 (PMC13141582; doi:10.1016/j.dialog.2026.100303)
Supplement: Supplementary material 1 — Interview guide [file mmc1.docx]

**Interview Guide**

**Study Title:** “Determinants of nutritional status and the effectiveness of Malnutrition Treatment Module for the prevention and control of malnutrition among young adolescent tribal girls of Ranchi, Jharkhand. A mixed method study.”

**Objective:** To explore determinants of nutritional status among adolescent tribal girls

**Key Informant**: Teachers of government schools in Kanke Block, Ranchi, Jharkhand.

**Methodology**: In-depth Interview

**Interview Questions**

Q1: Can you briefly describe yourself?

Q2: Can you briefly describe your role in this school?

Q3: How long have you been working with adolescent tribal students?

Q4: As a school teacher, what are your observations regarding the nutritional status of adolescent girls from tribal communities?

Q5: In your opinion, what are the major reasons for poor nutrition among adolescent girls?

Q6: In your experience how are sociodemographic factors influencing the health of adolescents?

Q7: In your opinion what are the common barriers or challenges faced by the adolescent girls to obtain good health?

Q8: What cultural or social practices influence their eating habits?

Q9: Do economic conditions affect food availability in households?

Q10: How do you monitor and evaluate the health of the adolescent girls at school?

Q11: How effective are school nutrition programmes such as the Mid-Day Meal programme, deworming and iron and folic acid supplementation?

Q12: What challenges do you face in implementing these programmes?

Q13: What barriers prevent adolescent girls from maintaining good nutrition?

Q14: Are there any gender-related factors affecting girls’ nutrition?

Q15: In your opinion, how can we improve the nutritional status of adolescent girls in this community?

Q16: Is there anything else you would like to share regarding adolescent nutrition in this community?
